# Spatiotemporal overlapping of dengue, chikungunya, and malaria infections in children in Kenya

**DOI:** 10.1186/s12879-023-08157-4

**Published:** 2023-03-29

**Authors:** Aslam Khan, Donal Bisanzio, Francis Mutuku, Bryson Ndenga, Elysse N. Grossi-Soyster, Zainab Jembe, Priscilla W. Maina, Philip K. Chebii, Charles O. Ronga, Victoria Okuta, A. Desiree LaBeaud

**Affiliations:** 1 Stanford University School of Medicine, Stanford, CA USA; 2 Center for Academic Medicine, 453 Quarry Road, Palo Alto, CA 94304 USA; 3 RTI International, Washington, DC, USA; 4 Technical University of Mombasa, Mombasa, Kenya; 5 Kenya Medical Research Institute, Kisumu, Kenya; 6 Msambweni County Referral hospital, Msambweni, Kenya

**Keywords:** Dengue virus, Chikungunya virus, Malaria, Children, Kenya

## Abstract

Malaria, chikungunya virus (CHIKV), and dengue virus (DENV) are endemic causes of fever among children in Kenya. The risks of infection are multifactorial and may be influenced by built and social environments. The high resolution overlapping of these diseases and factors affecting their spatial heterogeneity has not been investigated in Kenya. From 2014-2018, we prospectively followed a cohort of children from four communities in both coastal and western Kenya. Overall, 9.8% were CHIKV seropositive, 5.5% were DENV seropositive, and 39.1% were malaria positive (3521 children tested). The spatial analysis identified hot-spots for all three diseases in each site and in multiple years. The results of the model showed that the risk of exposure was linked to demographics with common factors for the three diseases including the presence of litter, crowded households, and higher wealth in these communities. These insights are of high importance to improve surveillance and targeted control of mosquito-borne diseases in Kenya.

**Supplementary Information:**

The online version contains supplementary material available at 10.1186/s12879-023-08157-4.

## Introduction

Acute febrile illness is one of the major reasons children seek medical care around the world, especially in tropical climates where Plasmodium spp. (malaria), dengue virus (DENV) and chikungunya virus (CHIKV) are endemic [1]. DENV has been identified as a significant cause of acute febrile illness worldwide and often disproportionately affects children with a more severe presentation of illness and higher proportion of mortality globally when compared to adults [2–4]. CHIKV has re-emerged as a significant cause of fever and there has been co-circulation demonstrated in areas with endemic DENV [5–7]. Malaria, the disease caused by infection from Plasmodium spp.*,*presents with variable symptomatology and is often implicated as a cause of fever in endemic regions, resulting in antiparasitic prescriptions [8–10]. For this article malaria will be used to refer to the pathogen, infection, and disease. Although DENV, CHIKV and malaria overlap in symptomatology, there are differences in transmission given the different associated vectors. Both CHIKV and DENV are transmitted by diurnal *Aedes aegypti* mosquitoes in contrast to malaria-transmitting nocturnal *Anopheles spp.*which mostly feed at night [11]. These pathogens are endemic in East Africa, where vector control efforts are often directed at malaria prevention, but our laboratory and others have shown there is a significant burden of *Aedes aegypti*, dengue, and chikungunya that should also be addressed [12–19].

There are multiple factors associated with transmission pertaining to the environment, socioeconomic status, urbanization, behavior, disease perception, and movement, among others [14, 20–22]. These structural and social factors, are both important contributors to vector proliferation and further transmission in endemic regions, and are important areas to address for disease mitigation by public health officials [23]. Specific diagnosis is challenging as there are often non-specific symptoms in mild disease, rendering misdiagnosis common if appropriate testing is not performed [8]. Furthermore, during outbreaks of CHIKV and DENV in Asia and the Americas, transmission clusters have been described in households with asymptomatic infected household members found to have viremia, suggesting the household may be a risk factor for acquiring infection [24–26]. Household preventive measures can reduce the risk of these mosquito-borne infections. In addition to household measures, community-level interventions can be implemented to control vectors such as container covers, use of mosquito repellants/insecticides, waste management, and house screening. Although arboviral infections have been associated with increased community waste, human crowding, rainfall, and baseline temperature, there are limited studies evaluating risk factors in areas with co-exposure to malaria and the environmental risk of infection in relation to both built and social environments [27, 28]. To assess whether household built and social environments are risk factors for infection, we mapped all positive cases for malaria, CHIKV, and DENV in our study sites in western and coastal Kenya and determined if exposure is clustered in space and what risk factors are associated with infection.

## Methods

### Recruitment, testing, and cohort survey

**Fig. 1:**
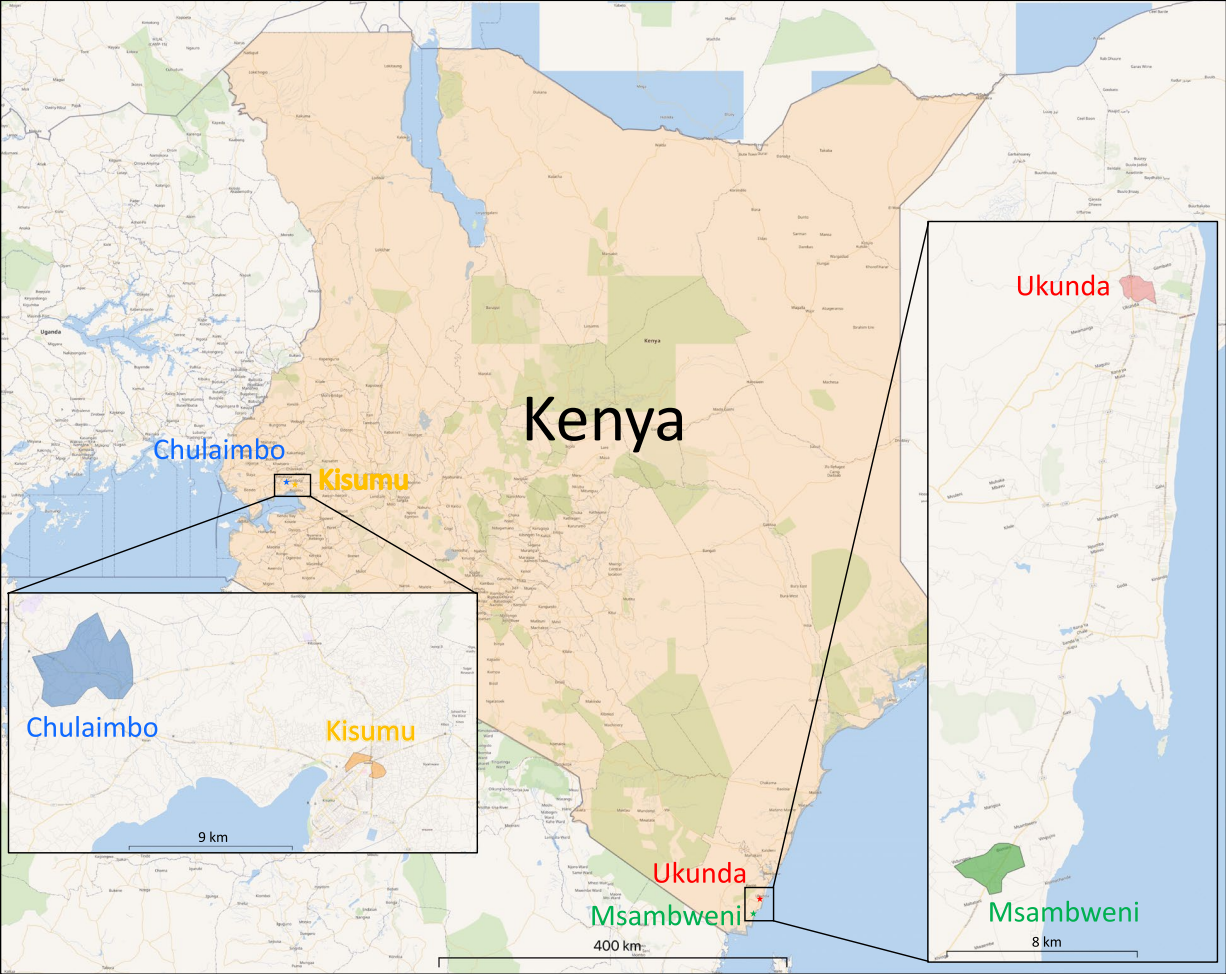
Study sites in western and coastal Kenya. The shaded regions near the city/village names are the respective zones for this study

Through an R01 (AI102918: PI: ADL) funded study, our team followed children 1-12 years of age for CHIKV and DENV exposure, measuring seroconversion over time in both densely and sparsely populated sites in Western Kenya and Coastal Kenya from 1/2014-12/2018 (Fig. 1). These locations were chosen in proximity to our collaborating institutions to reflect the community around Kisumu, Kenya, and Ukunda, Kenya, which are more densely populated city centers. The adjacent sites in Chulaimbo, Kenya, and Msambweni, Kenya are less densely populated and less developed in contrast to the respective city centers. Participants were recruited house to house in the region and consent was obtained by the parents/guardians. Verbal assent was obtained by children aged 7 years and older. The study site zones were chosen to represent the local community in the respective areas. The project was approved by the Stanford (31488) and KEMRI (SSC 2611) institutional review boards. Blood was collected and tested for DENV IgG and CHIKV IgG by ELISA and for malaria through blood smear testing [12, 29]. The children were followed over 5 years with 6-month interval blood collection, serologic testing (DENV and CHIKV IgG), and malaria testing at follow-up visits. Malaria testing occurred at the field hospitals and ELISA IgG testing was conducted at the field hospitals with repeat confirmatory testing performed at Stanford University. Cut-off values on ELISA testing for identification of seropositive and seronegative results were determined using plaque-reduction neutralization test (PRNT)-confirmed serum samples for CHIKV and DENV. A positive was any sample that had an optical density at or above three times the value of the negative control and at least half of the positive control reading [12, 29]. Almost all samples were tested for DENV and CHIKV by IgG ELISA and for samples that had significant discrepancies between results at both sites, the result for repeat testing at Stanford University was used for this study. Demographic data as well as travel, GPS data, activity, symptoms, and household factors were collected during the initial visit and repeated at each follow-up visit approximately every 6 months (see Additional file 1). Follow-up visits were conducted door to door on occasion and at a central facility to interact with the study participants. Participants were able to enroll at any time during the five years of the study and could drop out at any time, with late enrollees undergoing fewer follow-up visits. There were dropouts in the cohort over time and all visits were scheduled regardless of symptoms, with the majority qualifying as healthy visits.

### Statistical analysis

#### SES analysis

We calculated a wealth score for everyone based on asset ownership and the physical characteristics of their home. We considered variables related to durable assets (e.g., radio, motor vehicle, television), house ownership and characteristics (e.g., number of rooms used for sleeping and building materials), and access to utilities and infrastructure (e.g. sanitation facility and source of water) [30]. The wealth score was estimated using Multi Correspondence Analysis (MCA) [31]. MCA is a multivariate method developed for exploring discrete quantitative values that can be used to calculate an index based on the amount of variance explained by each included variable. The index calculated using MCA is similar to the one obtained by using a principal component analysis (PCA) [31]. The wealth scores were calculated using the first MCA linear combination that explained the greater part of the data's variance, then used to divide the households into four ordinal groups (Poorest, Poor, Rich, Richest), based on quartiles.

#### Household crowding index

A household crowding index (HCI) was calculated per each household [32]. HCI was defined as the total number of co-residents per household divided by the total number of rooms. The calculated HCI was divided in three categories: low crowding as HCI <1, moderate crowding HCI 1–2, and high crowding HCI >2 residents per room.

#### Spatial analysis

Each study site was divided into squares with 100m (meter) resolution. The number of DENV, CHIKV, and malaria cases were calculated for each square with at least one household included in the cohort study. The Getis-Ord Gi* local spatial clustering test was used to investigate the spatial pattern of DENV, CHIKV, and malaria cases recorded in each square during the study period [33]. The results of the Gi* local spatial test were used to identify hotspot areas in each site. The significance of the computed Gi* was estimated by comparing observed values to the random case distribution (null hypothesis) by randomly re-assigning the weekly cases to the squares. The statistical significance calculation was based on 100,000 Monte Carlo randomizations (*p*<0.05, with Bonferroni correction). A Kendall’s concordance coefficient (W) was used to investigate the overlap of DENV, CHIKV, and malaria hotspots [34]. Kendall’s W measures concordance between the values of two variables and it ranges from +1 (complete agreement) to -1 (no agreement). We considered Kendall's W <0.6 as low overlapping, ≥0.6 and < 0.8 as good overlapping, and ≥0.8 as high overlapping when comparing spatial distribution of DENV, CHIKV, and malaria hotpots.

#### Regression analysis

Logistic regression based on a generalized linear model (GLM) was applied to identify those factors linked with occurrence of transmission hot-spots. The modeling approach was used to investigate the association between DENV, CHIKV, and malaria hot-spots and the characteristics of children (e.g., use of repellent, use of bednet, traveling) and their households (water sources, floor and roof materials, presence of window screens, presence of litter). The GLM was adjusted for type of settlement (rural and urban) and location (coastal or western Kenya) of cohort site. Model selection technique based on Akaike Information Criteria (AIC) was used to identify those variables providing best logistic regression model [35]. All the variables investigated with the logistic regression are reported in Table-S7 in the Additional file 1.

This study was conducted in collaboration with two field teams in Western and coastal Kenya. Under the guidance of the principal investigator the teams recruited, surveyed, and sampled the participants. Samples were tested in Kenya in the laboratory and re-tested at Stanford University. The primary authors retrospectively evaluated this data and analyzed as described in addition to drafting the manuscript.

#### Patient and public involvement

Prior to launching the study our research team held barazas with the local community members and leaders to receive community input, permission, and to discuss the goals of the study. Our local collaborators at each site were also members of the community and held barazas to inform the community members of the results of the study.

## Results

During the study period, 3,445 children were enrolled in the study cohort. The median age of the children at the first visit was 7 years of age (interquartile range [IQR]: 5-10 years of age), with 50.9% being females. Among the enrolled children, 717 (20.8%) children dropped after the first visit, 352 (10.2) after the second visit, 317 (9.2%) after the third visit, 313 (9.1%) after the fourth visit, and 1753 (50.9%) received from five to seven visits. The geographic characteristics of infection are reported in Table 1.

**Table 1:** Characteristics of children by pathogen and village

**Site**	**2014-2018** **number of malaria positive**^**a**^** (prevalence, number of tested children)**	**2014-2018** **number of DENV seropositive**^**b**^** (seropositive, number of tested children)**	**2014-2018** **number of CHIKV seropositive**^**b**^** (seropositive, number of tested children)**
**Chulaimbo**	**902 (40.9%, 2,203)**	**40 (4.5%, 883)**	**185 (20.9%, 884)**
**Kisumu**	**495 (18.1%, 2,747)**	**21 (2.6%, 808)**	**42 (5.2%, 808)**
**Msambweni**	**540 (23.1%, 2,336)**	**75 (10.2%, 736)**	**64 (8.7%, 735)**
**Ukunda**	**100 (3.3%, 3,070)**	**45 (4.4%, 1,016)**	**29 (2.9%, 1,017)**
**Total**	**2,037 (19.7%, 10,362)**	**181 (5.3%, 3,443)**	**320 (9.3%, 3,444)**

### DENV and CHIKV exposure and infection

**Fig. 2:**
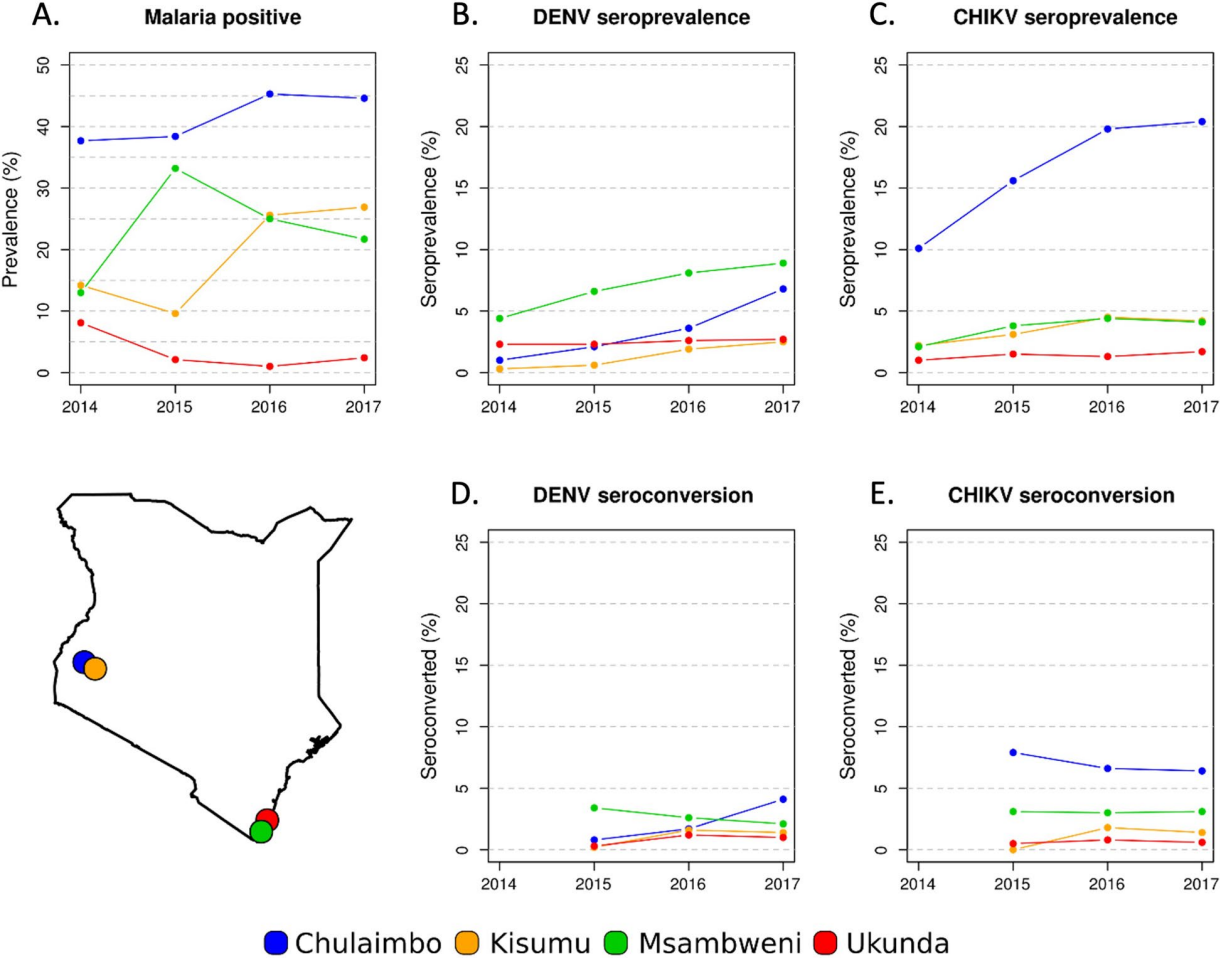
Trend of malaria prevalence, DENV and CHIKV seroprevalence and seroconversion. Trend of malaria prevalence, DENV, and CHIKV prevalence and seroconversions by village. **A** Malaria positivity trended in the cohort over time, **B** DENV seroprevalence trended over time with highest positivity found in Msambweni, **C** CHIKV seroprevalence trended over time with highest positivity found in Chulaimbo, **D** DENV seroconversions trended demonstrating stability over time, **E** CHIKV seroconversions trended over time demonstrating stability

From 2014 to 2018, 181 (5.3%) and 320 (9.3%) children tested positive for DENV and CHIKV, respectively (Table S1 and Table S2). The highest percentage of DENV seropositive children were recorded in Msambweni with a total of 75 cases (10.2%) (Table 1). Chulaimbo recorded the highest number of CHIKV seropositive children with a total of 185 cases (20.9%) (Table 1). DENV and CHIKV seroprevalence showed marked heterogeneity across the study area from 2014 to 2017 (Fig. 2). The seroprevalence of DENV increased in Chulaimbo, Kisumu, and Msambweni but remained constant in Ukunda over the study period (Fig. 2).

From 2014 to 2018, 107 (3.9%) and 174 (6.4%) children seroconverted for DENV and CHIKV, respectively. Among the cohort children, 28 (8.1%) individuals seroconverted for both DENV and CHIKV, suggesting exposure to both viruses during the study period. Msambweni was the site with the highest DENV seroconversion, with an overall 6.7% of seroconversions among follow-up children during the entire study period. Most seroconversions recorded in Msambweni occurred during 2015 (18.5%) and 2016 (12.6%) (Fig. 2). The other sites showed similar levels of seroconversions among cohort children during the study period (Fig. 2). DENV seroprevalence and seroconversion increased rapidly in Chulaimbo compared to the other three sites (Fig. 2). Msambweni was the only site in which the DENV seroconversion trend declined from 2015 to 2017 (Fig. 2). CHIKV seroconversions remained constant in Msambweni and Ukunda, resulting in a small increase of seroprevalence in the cohorts of the two sites (Fig. 2). The seroconversion trend in Kisumu showed an increase from 2015 to 2017, followed by a slight decline (Fig. 2).

**Fig. 3:**
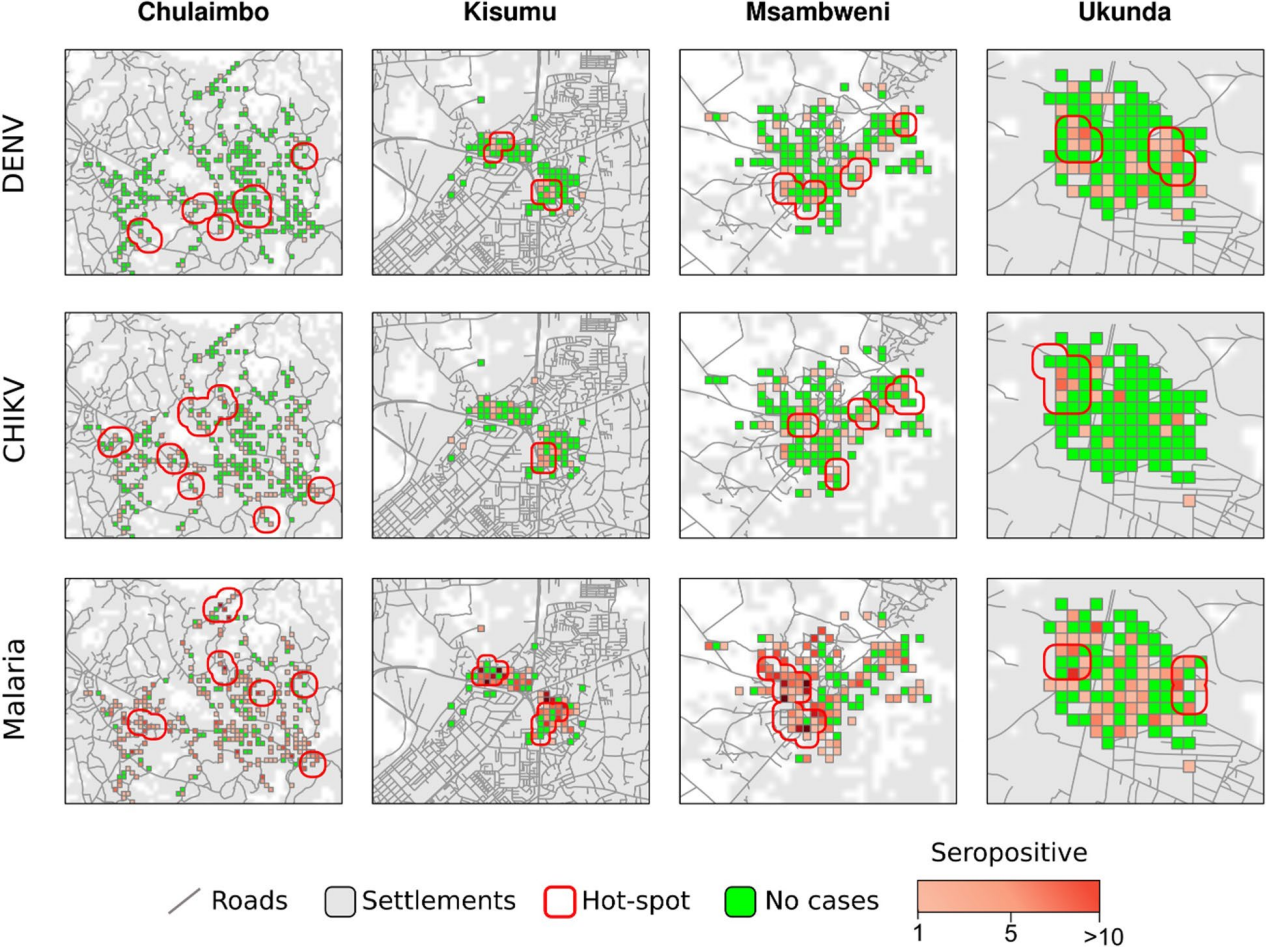
Spatial pattern of malaria prevalence, DENV and CHIKV seroprevalence. Each box represents 100m x 100m square area to reflect the maximum flight distance of the mosquito vectors. Statistically significant hotspots for these pathogens are outlined by the red lines with areas of overlap in the same region for the different pathogens

The spatial pattern of dengue and chikungunya cases had a marked spatial heterogeneity in all the sites (Fig. 3). The results of the Gi* local spatial test identified several significant hot-spots of DENV and CHIKV in all study locations.

### Malaria infections

From 2014 to 2018, 1,310 (38%) of all cohort children had a positive malaria blood smear (Table 1). Although some individuals did have rapid diagnostic testing, blood smears were the defining result used to confirm malaria infection in this study. During the study period, 14.6% (505 children) of tested children were positive for malaria in more than one year, equal to 38.5% of all malaria positive children. Cohorts in Chulaimbo, Kisumu, and Msambweni had ~40% of children infected by malaria from 2014 to 2018 (Table S5); the cohort in Ukunda recorded the lowest (9.1%) fraction of children with at least one positive malaria test (Table S5). The cohort in Chulaimbo had the highest mean annual prevalence (33.2%) among all cohorts with the Ukunda cohort reporting the lowest mean annual prevalence (3.1%) (Table S5). The highest malaria prevalence across the years was recorded in Chulaimbo (45.3%) during 2016 (Table S5). An increase of malaria prevalence was recorded in both western cohorts, Chulaimbo and Kisumu, while the coastal sites, Msambweni and Ukunda, showed a declining prevalence trend during the study period (Fig. 2). As seen for DENV and CHIKV, malaria prevalence showed a marked spatial heterogeneity across the study area from 2014 to 2017 (Fig. 3).

### Spatial analysis

The Getis-Ord local G test identified overlapping hot-spots of cases in all sites for the two arboviruses and malaria throughout the study period (Fig. 3). The Kendall’s concordance test highlighted that hot-spots of the three infections had strong overlap with some differences among the sites (Kendall’s W: median (MD)=0.69, interquartile range (IRQ)=0.47-0.75) (Table S6). Hot-spots identified in densely populated areas, Kisumu and Ukunda, had the highest level of overlapping (Kendall’s W: MD=0.75, IRQ=0.72-0.78) compared to the less densely populated settings, Chulaimbo and Msambweni (Kendall’s W: MD=0.46, IRQ=0.44-0.51) (Table S6). The overlap of DENV-CHIKV and CHIKV-malaria hot-spot dyads was higher in densely populated settings compared to less densely populated settings (Table S6). The DENV-malaria hot-spot dyad had high overlap in all sites beside Chulaimbo (Table S6). The highest level of overlapping was recorded for the DENV-malaria hot-spot dyad of Ukunda with a Kendall’s W=0.90 (*p*<0.01).

### Household characteristics associated to hot-spots

Presence of window screens, use of bednets, household crowding, roof material, presence of litter around the household, and the household wealth index were those variables included in the best model selected trough modelling selection analysis (Table S7). The association of the selected variables to the probability of the household to be in a transmission hot-spot was similar for the three mosquito-borne diseases (Table 2). Household crowding, presence of litter, and high wealth index were significantly associated with increased probability for a household to be in a transmission hotspot of DENV, CHIKV, and malaria (Table 2). Crowded households had a two-fold increased probability of being in a transmission hot-spot of CHIKV and malaria compared to non-crowded households (CHIKV: OR =2.02 [95% CI: 1.49-2.73], malaria: OR= 1.96 [95% CI: 1.47-2.61], *p*<0.05) (Table 2). The association of house crowding with being in a DENV hotspot (DENV: OR =1.68 [95% CI: 1.26-2.23]) was lower compared to being in a CHIKV or malaria hot-spot (Table 2). Litter left in household premises increased the risk of being in a transmission hot-spot by 29%, 65%, and 200% for DENV, CHIKV, and malaria, respectively (Table 2). Households with higher wealth had three times higher probability to be in a hot-spot of DENV and malaria and 69% higher probability to be in a CHIKV hot-spot compared to less wealthy households (Table 2). Window screens, metal roof, and use of a bednet protected the household from being in a transmission hot-spot (Table 2). The protection effect of window screen was stronger for malaria (OR =0.56 [95% CI: 0.43-0.73], *p*<0.05) compared to DENV (OR =0.80 [95% CI: 0.61-1.05], *p*=0.11) and CHIKV (OR =0.75 [95% CI: 0.56-0.99], *p*<0.05). Households with metal roofing had a significantly lower probability to be in transmission hot-spots for DENV, CHIKV, and malaria (Table 2). The protective effect of metal roof was lower for malaria (OR =0.32 [95% CI: 0.23-0.46], *p*<0.05) compared to DENV (OR =0.43 [95% CI: 0.29-0.63], *p*<0.05) and CHIKV (OR =0.32 [95% CI: 0.23-0.46], *p*<0.05). Bednet use was included in the best models and was associated, although not significantly, to a reduced probability of being in hot-spots for the three diseases (Table 2).

**Table 2:** Result of logistic regression used to identify factors linked to hot-spot occurrence. The table only shows variables included in the best model.

**Variable**	**DENV** **OR (95% CI)**	**CHIKV** **OR (95% CI)**	**Malaria** **OR (95% CI)**
**Window screens (ref. no screens)**	0.80 (0.61-1.05)	0.75 (0.56-0.99)*	0.56 (0.43-0.73)*
**Bednet ownership (ref. no bednets)**	0.72 (0.51-1.01)	0.76 (0.53-1.10)	0.81 (0.58-1.15)
**Crowed household (HCI>2) (ref. HCI</=2)**	1.68 (1.26-2.23)*	2.02 (1.49-2.73)*	1.96 (1.47-2.61)*
**Metal roof (ref. Natural materials)**	0.43 (0.29-0.63)*	0.66 (0.45-0.97)*	0.32 (0.23-0.46)*
**Litter presence (ref. no surrounding litter)**	1.29 (0.97-1.73)*	1.65 (1.21-2.27)*	2.04 (1.53-2.74)*
**High wealth index (ref. bottom three quartiles)**	3.16 (2.28-4.43)*	1.69 (1.21-2.38)*	3.36 (2.45-4.67)*

^*^Indicates findings that were statistically significant (*p*<0.05)

## Discussion

The areas of overlap in hotspots found in this study between DENV, CHIKV, and malaria along with similar associated risk factors suggests that both built and social environments carry significant influence in the transmission of these infections.

In this study we found higher seroconversions for CHIKV and DENV in the less densely populated study sites, compared to more densely populated study sites. Although this contrasts with prior studies suggesting more risk for arboviral infection in dense urban centers, this is similar to trends being reported where dengue seroprevalence has increased in less developed peri-urban and rural communities [36–38]. This change may be associated with alternative means of water collection when no piped water is available in those communities [37]. There are reports demonstrating the prevalence of the *Aedes spp*. vector for CHIKV/DENV transmission is more widespread than initially expected in the African continent [39, 40]. This spread can be exacerbated with the addition of pooling water for collection and storage, placing the population at increased risk for infection. Seroconversion rates in our cohorts remained relatively stable, suggesting constant CHIKV/DENV transmission during the study period. The hotspot associations were strongest in the densely populated sites with association of both the built and social environments contributing to transmission. Although these sites are relatively close together in proximity, there are significant differences between the communities, with the obvious difference being population density. As the communities become less dense, there is less piped water, less paved roads, less metal and tiled roofing, less window coverings, and more surrounding vegetation, muddy roads with pooled water, grass/natural roofing, and uninhabited land. The differences are rather substantial although the distances are not that far.

When evaluating risk factors, household crowding was significantly associated with being found in a hotspot. This could derive from an increased likelihood of someone from the household testing positive or individuals may serve as a reservoir for these pathogens and propagate transmission by local mosquitoes. Additionally household crowding can place an increased financial and resource strain on families, limiting use of bed nets or other protective measures for mosquito bites.

The presence of litter was also associated with being found in a hotspot for all three infections, with the strongest association for malaria [41]. Plastic containers and other waste can collect rainwater and have been shown to serve as breeding grounds for multiple mosquito species [15, 16, 23, 42–45]. A recent study in Brazil demonstrated increased seroprevalence of DENV and CHIKV in waste pickers at an open-air landfill [46]. The waste management infrastructure in western and coastal Kenya is changing but is reliant on households who often take their trash to a shared site as opposed to burning near their home. This association supports the importance of appropriate waste disposal/management for improved health.

“Higher wealth” was associated with being found in a transmission hot-spot for all three infections and can be representative of multiple factors. Given the finding contrasts to prior studies where poverty is a risk factor for arboviral infection, it is important to consider the overall wealth in our Kenyan study cohort is low with the highest wealth in those communities associated with being found in a transmission hotspot for malaria, DENV, and CHIKV. Although there are areas of overlap between the pathogens, this finding may be different if not incorporating all three pathogens with two different mosquito vectors. In comparing the differences between the sites, most of the participant families do not have means to purchase air conditioning, glass windows, automobiles, and even window screens. In this context, the higher wealth refers to those with more wealth in a very resource limited community and not necessarily absolute wealth as seen in other larger cities, where the discrepancy is much greater. When focusing on this community it is important to see there is a difference in risk for those with more means compared to the extremely poor, with ability to travel, increased exposure to others, and increased litter all being possible contributing factors. Higher wealth in this population may ultimately be linked to higher exposure due to behavioral factors and household characteristics that were not completely captured by the variables included in the study model.

The results of the study identified use of window screens, bed nets (although not significant), and metal roofing as protective factors that reduced the probability of transmission hotspot. Window screens can prevent mosquito travel into the home at day and night but bed nets are often used at night [47–49]. Assessing the effect of bed nets was limited by the widespread use of bed nets with a few children not using them. Additionally, there was no information if the communities experience high insecticide resistance which would change the efficacy of the bed nets. Typically the climate in our study sites is very warm and metal roof houses can generate significant heat, creating an unfavorable environment for mosquito survival [50]. Our laboratory has previously demonstrated that malaria transmission by *Anopheles gambiae* peaks at 25°C, whereas dengue transmission by *Aedes aegypti *peaks at 29°C [50, 51]. Metal roofing can generate indoor temperatures upwards of 35°C and has been shown to increase mosquito mortality, which can explain this finding [50].

Although there were significant associations found with the generalized linear model, there are a few limitations to this study. The viral infections and areas of clustering were all characterized by seroprevalence over specified time intervals and not PCR positivity, rendering it challenging to determine the true number of symptomatic and asymptomatic infections. The data was collected with interval follow-up visits and some participants dropped out of the study and new participants were recruited with less overall data points. The socioeconomic status was calculated with factors specific to our study cohort but there was limited wealth in the communities studied and the results may not be applicable to other populations. Given their relatively limited exposure to these pathogens compared to adults, we studied infection in children with some behavior and risk factors which may be different if studying adults.

Despite these limitations this study does provide further insight into mosquito-borne infections and highlights the seroprevalence trends for CHIKV and DENV over time in various communities in Kenya. The results also demonstrate the value of spatio-temporal modelling to better understand these infections. Similar type studies can be conducted in other communities where these infections are highly prevalent.

## Conclusions

Our findings demonstrate that there is significant overlapping with DENV, CHIKV, and malaria hotspots, especially in densely populated centers, with household crowding and surrounding litter as risk factors for being in transmission hotspots. The presence of window screens, bed nets, and metal roofing were protective, and households were less likely to be found in transmission hotspots, supporting that the built and social environment should be considered as a risk factor for transmission when addressing vector and infection control. The similar risk factors and protective factors for being found in transmission hotspots for all three infections can be targets to reduce the prevalence of these infections in the local communities. Kenyan public health officials and community members can utilize these findings to potentially reduce the burden of DENV, CHIKV, and malaria in their communities.

## Supplementary Information

**Additional file 1:** **Table S1.** Children who had DENV seropositive test in thestudy sites from 2014 to 2018.** Table S2.** Children who had CHIKVseropositive in the study sites from 2014 to 2018. **Table S3.** Childrenwho seroconverted for DENV in the study sites from 2014 to 2018.Thetables report the number of seroconverted children, the number of follow-upchildren, and the percentage of follow-up children who seroconverted. **TableS4.** CHIKV seroconversion in thestudy sites from 2014 to 2018. **Table S5.** Malaria positive childrenidentified in the study sites from 2014 to 2018.** Table S6.** Results fromKendall’s W comparing spatial pattern of DENV, CHIKV, and malaria hot-spots inthe three sites.** Table S7.** Variable included in the full model and thoseselected during model selection analysis.

## Data Availability

The datasets generated and/or analyzed during the current study are not publicly available due to ongoing studies and pending results involving this current data but are available from the corresponding author on reasonable request. They will become available when the pending studies have been completed.
