# Supplementary material for: Spatiotemporal overlapping of dengue, chikungunya, and malaria infections in children in Kenya
Source: BMC Infect Dis. 2023 Mar 29;23:183. doi: 10.1186/s12879-023-08157-4 (PMC10053720; doi:10.1186/s12879-023-08157-4)
Supplement: Supplementary file 1 — Additional file 1: Table S1. Children who had DENV seropositive test in thestudy sites from 2014 to 2018. Table S2. Children who had CHIKVseropositive in the study sites from 2014 to 2018. Table S3. Childrenwho seroconverted for DENV in the study sites from 2014 to 2018.Thetables report the number of seroconverted children, the number of follow-upchildren, and the percentage of follow-up children who seroconverted. TableS4. CHIKV seroconversion in thestudy sites from 2014 to 2018. Table S5. Malaria positive childrenidentified in the study sites from 2014 to 2018. Table S6. Results fromKendall’s W comparing spatial pattern of DENV, CHIKV, and malaria hot-spots inthe three sites. Table S7. Variable included in the full model and thoseselected during model selection analysis. [file 12879_2023_8157_MOESM1_ESM.pdf]

Table S1. Children who had DENV seropositive test in the study sites from 2014 to 2018.

Site	2014 number seropositive[a] (% seropositive, number of tested children)	2015 number seropositive[a] (% seropositive, number of tested children)	2016 number seropositive[a] (% seropositive, number of tested children)	2017 number seropositive[a] (% seropositive, number of tested children)	2018 number seropositive[a] (% seropositive, number of tested children)	2014-2018 number seropositive[a] (% seropositive, number of tested children)
Chulaimbo	6 (1.0%, 624)	13 (2.1%, 625)	18 (3.6%, 498)	30 (6.8%, 438)	2 (40%, 5)	40 (4.5%, 883)
Kisumu	2 (0.3%, 578)	4 (0.6%, 616)	11 (1.9%, 579)	13 (2.5%, 524)	6 (1.3%, 448)	21 (2.6%, 808)
Msambweni	27 (4.4%, 617)	42 (6.6%, 632)	44 (8.1%, 541)	45 (8.9%, 508)	6 (17.1%, 35)	75 (10.2%, 736)
Ukunda	16 (2.3%, 691)	14 (2.3%, 606)	18 (2.6%, 683)	17 (2.7%, 634)	11 (2.4%, 460)	45 (4.4%, 1016)
Total	51 (2%, 2510)	73 (2.9%, 2479)	91 (4%, 2301)	105 (5%, 2104)	25 (2.6%, 948)	181 (5.3%, 3,443)

[a]children who had at least one positive test (ELISA, DENV IgG)

Table S2. Children who had CHIKV seropositive in the study sites from 2014 to 2018.

Site	2014 number seropositive[a] (% seropositive, number of tested children)	2015 number seropositive[a] (% seropositive, number of tested children)	2016 number seropositive[a] (% seropositive, number of tested children)	2017 number seropositive[a] (% seropositive, number of tested children)	2018 number seropositive[a] (% seropositive, number of tested children)	2014-2018 number seropositive[a] (% seropositive, number of tested children)
Chulaimbo	63 (10.1%, 624)	98 (15.8%, 622)	100 (20.2%, 496)	90 (20.8%, 432)	0 (0%, 5)	185 (20.9%, 884)
Kisumu	13 (2.2%, 579)	19 (3.1%, 616)	26 (4.5%, 577)	22 (4.2%, 524)	17 (3.8%, 446)	42 (5.2%, 808)

Msambweni	13 (2.1%, 612)	24 (3.8%, 630)	24 (4.4%, 543)	21 (4.1%, 508)	2 (5.7%, 35)	64 (8.7%, 735)
Ukunda	7 (1.0%, 691)	9 (1.5%, 607)	9 (1.3%, 682)	11 (1.7%, 634)	11 (2.4%, 458)	29 (2.9%, 1,017)
Total	96 (3.8%, 2506)	150 (6.1%, 2475)	159 (6.9%, 2298)	144 (6.9%, 2098)	30 (3.2%, 944)	320 (9.3%, 3,444)

[a]children who had at least one positive test (ELISA, CHIKV IgG)

Table S3. Children who seroconverted for DENV in the study sites from 2014 to 2018. The tables report the number of seroconverted children, the number of follow-up children, and the percentage of follow-up children who seroconverted.

Site	2015 number seroconverted[a] (% seroconverted[b], number of tests[c])	2016 number seroconverted[a] (% seroconverted[b], number of tests[c])	2017 number seroconverted[a] (% seroconverted[b], number of tests[c])	2018 number seroconverted[a] (% seroconverted[b], number of tests[c])	2014-2018 number seroconverted[d](% seroconverted[e], number follow-up children[f])
Chulaimbo	5 (0.6%, 843)	8 (0.9%, 856)	17 (2.2%, 771)	0 (0%, 4)	30 (4.8%, 630)
Kisumu	1 (0.2%, 419)	9 (0.9%, 991)	7 (0.8%, 913)	0 (0%, 434)	17 (2.6%, 661)
Msambweni	19 (2%, 939)	13 (1.5%, 886)	10 (1.4%, 705)	0 (0% 33)	42 (6.7%, 627)
Ukunda	2 (0.5%, 415)	8 (0.9%, 853)	6 (0.6%, 1072)	2 (0.4%, 451)	18 (2.2%, 809)
Total	27 (1%, 2,616)	38 (1.1%, 3,586)	40 (1.2%, 3,461)	2 (0.2%, 922)	107 (3.9%, 2,727)

[a]Number of children who seroconverted after negative test; [b] Percentage of children who seroconverted after negative test among follow-up children interviewed during the year; [c]Number of follow-up children who never tested positive for DENV during the previous years; [d]Number of children who seroconverted after negative test at least once during the study period; [e]Percentage among follow-up children interviewed during the whole study period with negative first test; [f]Number of follow-up children interviewed during the whole study period with negative first test.

Table S4. CHIKV seroconversion in the study sites from 2014 to 2018

Site	2015 number seroconverted[a] (% seroconverted[b], number follow-up children[c])	2016 number seroconverted[a] (% seroconverted[b], number follow-up children[c])	2017 number seroconverted[a] (% seroconverted[b], number follow-up children[c])	2018 number seroconverted[a] (% seroconverted[b], number follow-up children[c])	2014-2018 number seroconverted[d](% seroconverted[e], number follow-up children[f])
Chulaimbo	38 (4.5%, 843)	27 (3.3%, 818)	24 (3.4%, 705)	0 (0%, 4)	89 (14.1%, 625)
Kisumu	0 (0%, 419)	10 (1%, 992)	7 (0.8%, 917)	2 (0.5%, 436)	19 (2.9%, 661)
Msambweni	18 (1.9%, 939)	15 (1.7%, 885)	15 (2.1%, 706)	1 (3%, 33)	49 (7.8%, 625)
Ukunda	3 (0.7%, 415)	4 (0.6%, 853)	3 (0.4%, 1070)	5 (1.1%, 448)	17 (2.1%, 800)
Total	59 (2.3%, 2616)	57 (1.6%, 3548)	50 (1.5%, 3398)	8 (0.9%, 921)	174 (6.4%, 2719)

[a]Number of children who seroconverted after negative test; [b] Percentage of children who seroconverted after negative test among follow-up children interviewed during the year; [c]Number of follow-up children who never tested positive for DENV during the previous years; [d]Number of children who seroconverted after negative test at least once during the study period; [e]Percentage among follow-up children interviewed during the whole study period with negative first test; [f]Number of follow-up children interviewed during the whole study period with negative first test.

Table S5. Malaria positive children identified in the study sites from 2014 to 2018.

Site	2014 number positive[a] (% positive, number of tested children)	2015 number positive[a] (% positive, number of tested children)	2016 number positive[a] (% positive, number of tested children)	2017 number positive[a] (% positive, number of tested children)	2018 number positive[a] (% positive, number of tested children)	2014-2018 number positive[a] (% positive, number of tested children)
Chulaimbo	235 (37.7%, 624)	241 (38.4%, 627)	229 (45.3%, 505)	197 (44.6.8%, 442)	0 (0%, 5)	353 (40.9%, 884)
Kisumu	82 (14.2%, 579)	59 (9.6%, 616)	148	141	65	368 (45.5%, 808)

			(25.6%, 577)	(26.9%, 524)	(14.5%, 446)	
Msambweni	80 (13%, 617)	210 (33.2%, 633)	136 (25%, 543)	110 (21.7%, 508)	4 (11.4%, 35)	318 (43.2%, 736)
Ukunda	56 (8.1%, 691)	13 (2.1%, 608)	7 (1%, 683)	15 (2.4%, 634)	9 (2%, 460)	93 (9.1%, 1,017)
Total	453 (18%, 2,511)	523 (21%, 2,485)	520 (22.5%, 2,310)	463 (22%, 2,108)	78 (8.2%, 948)	1,310 (38%, 3,334)

[a]children who had at least one positive RDT

Table S6. Results from Kendall's W comparing spatial pattern of DENV, CHIKV, and malaria hot-spots in the three sites.

**Site**	**DENV-CHIK-malaria (Kendall's W)**	**DENV-CHIK (Kendall's W)**	**DENV- malaria (Kendall's W)**	**CHIK-malaria (Kendall's W)**
Chulaimbo	0.35	0.45	0.48	0.57*
Kisumu	0.68*	0.77*	0.79*	0.70*
Msambweni	0.41*	0.45	0.71*	0.49
Ukunda	0.78*	0.73*	0.90*	0.74*

Table S7. Variable included in the full model and those selected during model selection analysis.

Variable	DENV	CHIKV	Malaria
Household performs mosquito control activities	-	-	-
Use of coil in the household	-	-	-
Use of repellents in the household	-	-	-
House ownership	-	-	-
House floor type	-	-	-
House roof type	-	-	-
House cooking fuel	-	-	-
House access to electricity	-	-	-
Household toilet type	-	-	-
Household type of water source	-	-	-
Household has livestock	-	-	-

**Presence of buckets around the household**	-	-	-
**Child used repellent**	-	-	-
**Child uses coil**	-	-	-
**Child sleeps under bednet**	-	-	-
**Child has travelled outside the site**	-	-	-
**Child has played outside**	-	-	-
Selected in at least one model			
**Window screens**	+	+	+
**Bednet ownership**	+	+	+
**Household crowding**	+	+	+
**Metal roof (ref. Natural materials)**	+	+	+
**Fraction children with travel history**	+	+	+
**Litter presence**	+	+	+

**High SES**	+	+	+
